# High Prevalence, Genetic Diversity and Intracellular Growth Ability of *Legionella* in Hot Spring Environments

**DOI:** 10.1371/journal.pone.0059018

**Published:** 2013-03-18

**Authors:** Tian Qin, Gebin Yan, Hongyu Ren, Haijian Zhou, Huanxin Wang, Ying Xu, Mingqiang Zhao, Hong Guan, Machao Li, Zhujun Shao

**Affiliations:** 1 State Key Laboratory for Infectious Disease Prevention and Control, National Institute for Communicable Disease Control and Prevention, Chinese Center for Disease Control and Prevention, Changping, Beijing, China; 2 Changping District Center for Disease Control and Prevention, Changping, Beijing, China; University of Louisville, United States of America

## Abstract

**Background:**

*Legionella* is the causative agent of Legionnaires' disease, and hot springs are a major source of outbreaks of this disease. It is important from a public health perspective to survey hot spring environments for the presence of *Legionella*.

**Methods:**

Prospective surveillance of the extent of *Legionella* pollution was conducted at three hot spring recreational areas in Beijing, China in 2011. Pulsed-field gel electrophoresis (PFGE) and sequence-based typing (SBT) were used to describe the genetic polymorphism of isolates. The intracellular growth ability of the isolates was determined by interacting with J774 cells and plating the dilutions onto BCYE agar plates.

**Results:**

Overall, 51.9% of spring water samples showed *Legionella-*positive, and their concentrations ranged from 1 CFU/liter to 2,218 CFU/liter. The positive rates of *Legionella* were significantly associated with a free chlorine concentration of ≥0.2 mg/L, urea concentration of ≥0.05 mg/L, total microbial counts of ≥400 CFU/ml and total coliform of ≥3 MPN/L (p<0.01). The *Legionella* concentrations were significantly associated with sample temperature, pH, total microbial counts and total coliform (p<0.01). *Legionella pneumophila* was the most frequently isolated species (98.9%), and the isolated serogroups included serogroups 3 (25.3%), 6 (23.4%), 5 (19.2%), 1 (18.5%), 2 (10.2%), 8 (0.4%), 10 (0.8%), 9 (1.9%) and 12 (0.4%). Two hundred and twenty-eight isolates were analyzed by PFGE and 62 different patterns were obtained. Fifty-seven *L. pneumophila* isolates were selected for SBT analysis and divided into 35 different sequence types with 5 main clonal groups. All the 57 isolates had high intracellular growth ability.

**Conclusions:**

Our results demonstrated high prevalence and genetic polymorphism of *Legionella* in springs in Beijing, China, and the SBT and intracellular growth assay results suggested that the *Legionella* isolates of hot spring environments were pathogenic. Improved control and prevention strategies are urgently needed.

## Introduction

Legionellosis is an environment-related, acute respiratory infection that is caused by gram-negative bacteria of the genus *Legionella*. *Legionella* widely exist in environmental waters, and they are particularly prevalent in warm water. To date, more than 50 *Legionella* species have been characterized, and 25 species are known to cause human disease [1], [2]. Most human infections are caused by *Legionella pneumophila*, which is responsible for approximately 90% of the identified clinical cases, and the predominant serogroup is serogroup 1 [1], [3], [4], [5].

Transmission of bacteria from the environment to humans occurs via inhalation or aspiration of *Legionella*-containing aerosols [6], [7]. The water systems of artificial facilities, including potable water systems [8], [9], spa water [10] and cooling towers [11], [12] in large buildings, hotels, hospitals and public baths that are contaminated by *Legionella* are the implicated sources of outbreaks and sporadic cases of Legionnaires' disease. Thus, it is important from a public health perspective to continually survey environmental water systems for the presence of *Legionella* [13], [14], [15], [16]. In particular, the contamination of hot-water systems has gradually become recognized as an important risk factor all over the world [17], [18], [19], [20], [21], [22], and many cases of Legionnaires' disease have been detected in *Legionella*-contaminated hot-water systems.

In recent years, hot springs have been recognized as a major source of outbreaks of legionellosis. Several outbreaks and a number of cases of *Legionella* infection that were associated with hot springs have been reported in Japan, Spain and France [23], [24], [25]. In 2002, a large outbreak of legionellosis that affected two hundred and ninety-five patients occurred in a hot spring in Japan [24], [26]. In China, hot springs have become an increasingly popular form of recreation, and many people enjoy bathing in hot spring tubs. It is important to survey the contamination of *Legionella* in hot springs where people bath to avoid serious outbreaks of Legionnaires' disease. In our previous study that compared the ability of four methods (i.e., culture, PCR, quantitative real-time PCR and EMA-quantitative real-time PCR) to detect *Legionella* in different types of water systems, we isolated *Legionella* from spring water samples at a rate of up to 93.3% by quantitative real-time PCR. When using the culture method, the percent-positive rate for *Legionella* was 54.4%, and the concentrations of *Legionella* were as high as 1,109 CFU/liter [27]. These results showed high degree of pollution of *Legionella* in spring water samples in China. However, that study did not systematically focus on the *Legionella* in springs, and the spring water samples were only used to compare the detection ability of different methods.

The aim of our study is to detect *Legionella* in hot spring water systems by collecting samples for one year from three hot spring recreational areas in Beijing, China so that we may ascertain whether factors such as temperature, pH and total microbial counts are related to the extent of *Legionella* pollution. The serogroup of *Legionella* isolates were determined. Genetic characteristics were analyzed using pulsed-field gel electrophoresis (PFGE) and sequence-based typing (SBT) methods. Furthermore, the intracellular growth ability of the isolates was determined.

## Materials and Methods

### Sample collection

We selected three hot spring recreational areas in Beijing as study points. At each area, five spa pools were selected for sampling every month in 2011 except in May, and one to four samples were collected from each spa pool based on the size of the pools. All 308 spa water samples were collected during the one-year study period, and the procedure for collection and pretreatments of the environmental water samples was based on the protocol according to ISO 11731 (1998). Five hundred milliliters of each water sample was collected from a reservoir or condensation pan and placed in a sterile, screw-capped container. Chlorine in the water samples was inactivated by the addition of sodium thiosulfate.

### Ethics statements

Water sample collection in this study was carried out on private lands, and we confirm that the owners of the lands gave permission to conduct the study on these sites. No specific permissions were required for these locations/activities, because there are no endangered or protected species in these hot spring recreational areas and this study did not involve any endangered or protected species.

### *Legionella* detection

To detect *Legionella*, 200 microliters of the water samples were centrifuged at 6,000 g for 10 min in a 300 ml-capacity centrifuge bottle. The pellets were resuspended in 3 ml of distilled water, and the resuspended pellets were serially diluted 10-fold with sterile water. Diluted and undiluted samples (100 µl each) were plated onto GVPC agar (Oxoid, Hampshire, United Kingdom), and the plates were incubated at 37 °C for 10 days. Colonies were identified as *Legionella* using the L-cysteine requirement test, Gram staining and slide agglutination using polyclonal antisera (Denka Seiken, Tokyo, Japan).

### Physical-chemical and microbiological analyses of water samples

Water temperature and its pH were measured in situ using a portable pH meter (D-24E, Horiba Co., Japan). The samples were transported to the lab immediately. The residual free chlorine concentration was assayed using DPD liquid (HACH, Loveland, USA). The urea concentration was measured using the Standard Method of the Chinese National Institute of Standards (GB/T18204-2000).

The total microbial counts at 36 °C were obtained twice by the pour-plate method on plate count agar (Oxoid, Hampshire, United Kingdom). The plates were incubated at 36 °C for 48 h, and total coliform was measured by membrane filtration procedures.

### PFGE

We used the 1-day, standardized PFGE protocol for *L. pneumophila* [28]. Cell suspensions were placed in polystyrene tubes (Falcon; 12×75 mm), and their optical density was adjusted to 3.8–4.0 using a Densimat photometer (BioMérieux, Marcy l'Etoile, France). *Legionella* slices were digested using 30 U per slice of *Asc*I (New England Biolabs, Ipswich, MA, USA) for 4 h at 37 °C, and electrophoresis was performed using a CHEF-DRIII system (Bio-Rad Laboratories, Hercules, CA, USA). Electrophoresis was run with a switch time of 6.8 s to 54.2 s for 19 h, and images were captured using a Gel Doc 2000 system (Bio-Rad) and converted to TIFF files. The TIFF files were analyzed using the BioNumerics version 5.1 software (Applied Maths, Kortrijk, Belgium). Similarity analysis of the PFGE patterns was performed by calculating the Dice coefficients (SD) [29], with customized tolerance for each EP, and clustering was created using the unweighted-pair group method with average linkages (UPGMA).

### SBT and allelic diversity analyses

Genotyping was conducted via the standard, sequence-based typing (SBT) method of the European Working Group for *Legionella* Infections (EWGLI) using 7 genes (*flaA*, *pilE*, *asd*, *mip*, *mompS*, *proA* and *neuA*) [30], [31]. The SBT database that was available on the EWGLI website (http://www.ewgli.org/) was used for nucleotide analysis, and the sequences were compared with those in the SBT database, which were also available on the website (http://www.hpa-bioinformatics.org.uk/legionella/legionella_sbt/php/sbt_homepage.php). Additionally, sequence types (STs) that were not available in the EWGLI SBT database were represented alphanumerically in this study (e.g., ST-CN1).

The clonal complexes were analyzed using the eBURST V3 software (http://eburst.mlst.net), and clusters of related STs that descended from a common ancestor were defined as clonal groups (CGs). Single genotypes that corresponded to no CG were defined as singletons, and the BioNumerics software was used to create a minimum spanning tree. In the minimum spanning tree, the founder ST was defined as the ST with the greatest number of single-locus variants. Types are represented by circles, and the size of a circle indicates the number of strains of this particular type. Heavy solid lines connect two types that differ within a single locus; light solid lines connect double-locus variants; heavy dotted lines connect triple-locus variants; light dotted lines connect quadruple-locus variants; and gray circles represent STs that are not part of any clonal complex.

### Intracellular growth assay

The isolated *Legionella* strains were grown in BYE broth until they reached early stationary phase. Approximately 2×10^9^ bacteria were pelleted, resuspended and diluted (1∶1,000) in RPMI 1640 tissue culture medium. The bacteria were then added to J774 cells (2×10^5^ per well) in 24-well dishes to give a multiplicity of infection (MOI) of approximately 10. The infected cells were incubated at 37 °C under 5% CO_2_-air for 1.5 h and washed three times with PBS to remove extracellular bacteria. To measure bacterial internalization, 1 ml of sterile, distilled water was added to the wells to release intracellular bacteria from the host cells, and the CFUs were determined by plating dilutions on BCYE agar plates. To each of the wells, 0.5 ml of fresh tissue culture medium was added, and the intracellular and extracellular bacteria in each well were combined at 24-h intervals. The total number of CFUs was determined by plating the dilutions onto BCYE agar plates.

The J774 cell monolayers were prepared on cover slips by the same procedures as described above. The cells were infected with *L. pneumophila* philadelphia-1 and the other isolated strains. After 48 h of infection, the infected J774 cells were stained by Gimenez staining and observed under a light microscope.

### Statistical analysis

Data were analyzed using SPSS for the Windows Release 11.0.1 software (SPSS Inc, Chicago, IL, USA). The chi square test or Fisher exact test were used for analyzing qualitative data, while the t test or Mann-Whitney test were used to analyze quantitative data. The odds ratios (OR) and 95% confidence interval (CI) were calculated to assess categorical risk variables associated with *Legionella*-positive test results. The bacterial concentrations of *L. pneumophila* in intracellular growth assay were analyzed by the t test. The results were considered statistically significant when the P value was <0.05.

## Results

### The degree of pollution of *Legionella* in spring water samples

All 308 spring water samples were tested, and *Legionella* was cultured from 160 of those samples. Viable counts for the positive samples ranged from 1 CFU/liter to 2,218 CFU/liter with a median of 97 CFU/liter. Overall, the data presented a positive rate of *Legionella* of 51.9% (160/308) in the spring water samples of this study. Of the samples, 37.5% were isolated over 100 CFU/liter, and 7.5% exceeded 1,000 CFU/liter.

All of the 15 studied spa pools were positive for *Legionella* in at least one sample during the one-year study period. In January, all 15 spa pools were positive for *Legionella*, and in June, only six spa pools were positive for *Legionella*. The monthly average *Legionella* viable contents for each pool ranged from 0.5 CFU/liter to 569 CFU/liter, and two pools exhibited a monthly average concentration of *Legionella* that was higher than 100 CFU/liter.

### Site and seasonal variations in the level of *Legionella*

The isolation rates and concentrations of *Legionella* were different between the three hot spring recreational areas. The isolation rates were 85.5%, 45.5% and 43.6% in areas A, B and C, respectively. The average bacterial counts of *Legionella* in each month at area A ranged from 0.3 to 829 CFU/liter, and at areas B and C, the average counts were 0–52 CFU/liter and 0–104 CFU/liter, respectively. The isolation rate and concentration of *Legionella* from area A were significantly higher than those of areas B and C (P<0.01).

**Figure 1:**
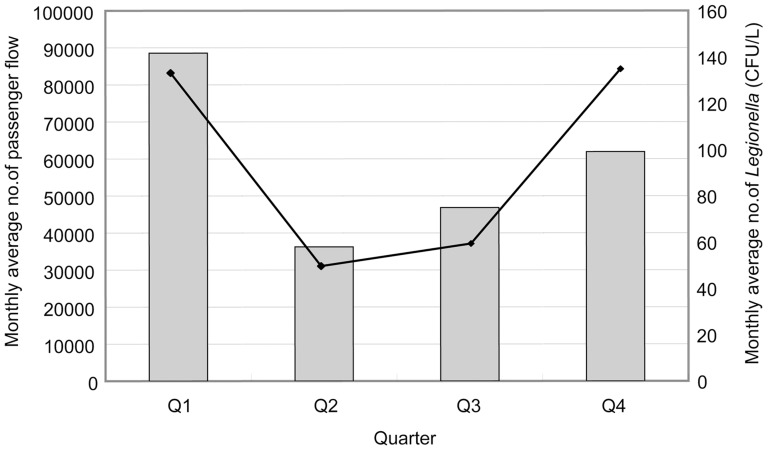
Relationship between the monthly average number of *Legionella* and monthly average number of passenger flow. The curve represents the monthly average number of *Legionella* (CFU/L), and the histogram represents the monthly average number of passenger flow.

The isolation rates and concentrations of *Legionella* were higher during the spring (73.3%, 132 CFU/liter) and winter (62.2%, 134 CFU/liter) than in summer (50.0%, 49 CFU/liter) or autumn (48.9%, 58 CFU/liter) (Figure 1). Figure 1 also shows a positive association between *Legionella* concentrations and passenger flow. In spring and winter, the numbers of passengers visiting hot spring recreational areas were higher than in summer and autumn.

### Correlation of the occurrence of *Legionella* with water quality parameters

We have measured the temperature, pH, free chlorine concentration and urea concentration of every spring water sample that was obtained. The average temperature and pH ranged from 31 °C to 49 °C and 7.80 to 8.04, respectively. No significant association was found between the *Legionella* positive rate and sample temperature ≥40 °C (OR = 1.820, p = 0.06) or pH≥7.9 (OR = 0.685, p = 0.286) (Table 1). However, the *Legionella* concentration was significantly associated with the sample temperature (correction coefficient  =  0.226, p<0.01) and pH (correction coefficient  =  −0.349,p<0.01). *Legionella* colonization was positively associated with a free chlorine concentration of ≥0.2 mg/L or urea concentration of ≥0.05 mg/L, but no significant association was found between the *Legionella* concentration and free chlorine or urea concentration (p = 0.135 and p = 0.078, respectively).

**Table 1:** Association of physical, chemical, and microbiological water characteristics with *Legionella* spp. concentration of spa water samples.

Characteristic	No. (%) of positive samples for *Legionella*		Odds ratios (95% confidence interval)	P
	With characteristics	Without characteristics		
Sample Temp≥40 °C	56 (65.1%)	40 (50.6%)	1.820 (0.974–3.402)	0.060
pH≥7.9	65 (55.6%)	31 (64.6%)	0.685 (0.342–1.373)	0.286
Urea ≥0.05 mg/L	17 (37.8%)	69 (75.8%)	0.315 (0.155–0.642)	0.001
Free chlorine ≥0.2 mg/L	4 (13.3%)	92 (68.1%)	0.072 (0.024–0.219)	0.000
Total plate count ≥400 CFU/L	72 (71.3%)	24 (37.5%)	4.138 (2.129–8.044)	0.000
*E. coli* count ≥3 MPN/L	72 (74.2%)	24 (35.3%)	5.280 (2.691–10.361)	0.000

The microbiological parameters of the water samples that were examined included the total microbial counts and total coliform. Sixty percent of the tested water samples showed total microbial counts ≥400 CFU/ml and total coliform ≥3 MPN/L. A significant association was found between the positive rate of *Legionella* and total microbial counts ≥4001 CFU/ml or total coliform ≥3 MPN/L. The *Legionella* concentration was also found to be associated with the total microbial counts (correction coefficient = 0.185, p<0.01) and total coliform (correction coefficient  = 0.298, p<0.01).

### Distribution of serogroups of *Legionella* isolates

We selected one to five isolates from each water sample to determine their species and serogroups. In total, 268 isolates of *Legionella* from 160 samples were examined. To our surprise, of the 160 *Legionella*-positive samples, two or more *Legionella* species or serogroups were identified from 70 samples (43.8%). In nine samples, strains belonging to four or five different *L. pneumophila* serogroups were detected, and these cases were observed in all three hot spring recreational areas.

**Figure 2:**
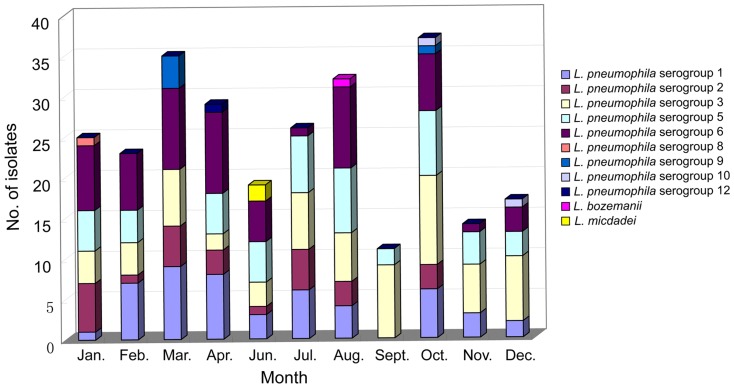
Distribution of *Legionella* species and serogroups from springs in Beijing, China.

*L. pneumophila* was the most frequently isolated species and made up 98.9% of the strains that were isolated. Two hundred and sixty-five isolates were identified as *L. pneumophila*, and the serogroups included serogroups 3 (67 isolates, 25.3%), 6 (62, 23.4%), 5 (51, 19.2%), 1 (49, 18.5%), 2 (27, 10.2%), 8 (1, 0.4%), 10 (2, 0.8%), 9 (5, 1.9%) and 12 (1, 0.4%). Three isolates were determined to be non-*L. pneumophila*: one was *Legionella bozemanii* and two were *Legionella micdadei* (Figure 2, Table 2). However, the relative proportion of the isolates of these serogroups varied within the three areas. *L. pneumophila* serogroup 3 was predominant in area A, where the other four serogroups (serogroup 1, 2, 5 and 6) were also common. *L. pneumophila* serogroup 6 strains were the second predominant group in area A and the most predominant in area B (37.3%), but no *L. pneumophila* serogroup 6 strains were isolated from area C. In area C, *L. pneumophila* serogroup 5 strains were predominant and accounted for 48.6% of the isolates.

**Table 2:** The number of spa water samples, spa pools and months contaminated by different *Legionella* species and serogroups.

serogroups	spa resort A			spa resort B			spa resort C			Total		
	No. of samples	No. of pools	No. of months	No. of samples	No. of pools	No. of months	No. of samples	No. of pools	No. of months	No. of samples	No. of pools	No. of months
Lp sg1	37	5	8	5	2	3	7	4	4	49	11	10
Lp sg2	24	5	7	3	1	3				27	6	8
Lp sg3	49	5	11	9	4	6	9	5	4	67	14	11
Lp sg5	28	4	8	6	3	6	17	5	6	51	12	10
Lp sg6	43	5	9	19	5	7				62	10	10
Lp sg8							1	1	1	1	1	1
Lp sg9				5	3	2				5	3	2
Lp sg10				1	1	1	1	1	1	2	2	2
Lp sg12	1	1	1							1	1	1
*L.bozemanii*				1	1	1				1	1	1
*L.micdadei*				2	2	1				2	2	1

*L. pneumophila* serogroup 3 was found most frequently and was isolated from 67 samples of 14 pools throughout the one-year study period. This group was followed in predominance by *L. pneumophila* serogroups 6, 5, 1 and 2 (Table 2). These serogroups were isolated for more than eight months; however, no *L. pneumophila* serogroup 2 or 6 strains were isolated from area C. Other *L. pneumophila* serogroups and non-*L. pneumophila* strains were isolated less frequently. For example, *L. pneumophila* serogroup 8 was only isolated from area C in January; *L. pneumophila* serogroup 12 was only isolated from area A in April; *L. pneumophila* serogroup 9 was isolated from area B in March and October; two *L. pneumophila* serogroup 10 strains were isolated from area B in December and from area B in October; and *L. micdadei* and *L.bozemanii* were only isolated from area B in June and August, respectively.

### PFGE and SBT analysis of *Legionella* isolates

**Figure 3:**
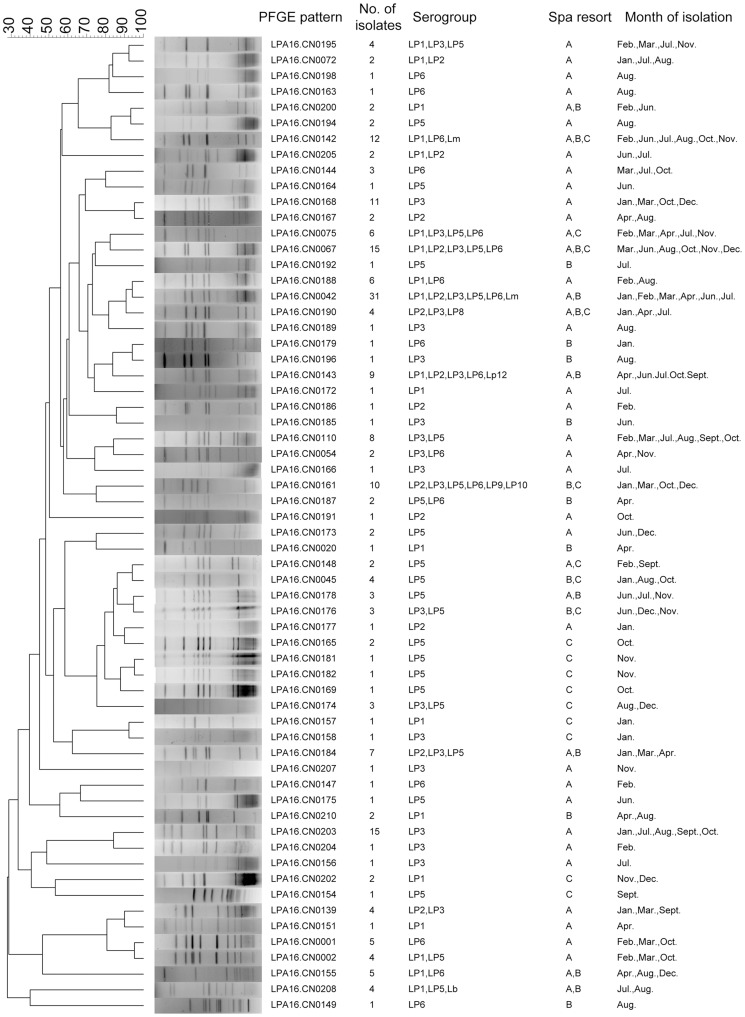
Clustering results of patterns obtained by PFGE analysis of 228 *Legionella* strains. These strains were isolated from three hot spring recreational areas in Beijing, China during the one-year study period.

Total 228 isolates were selected to study by PFGE, and 62 different patterns were obtained (Figure 3). The pattern LPA16.CN0042 was the most frequently occurring pattern and contained 31 strains that were isolated from three hot spring recreational areas over six months. Thirty-five patterns contained more than one isolate, and among these, 15, 21 and 32 patterns contained isolates from different hot spring recreational areas, serogroups and months, respectively. These results indicated a high genetic polymorphism among these tested isolates.

**Figure 4:**
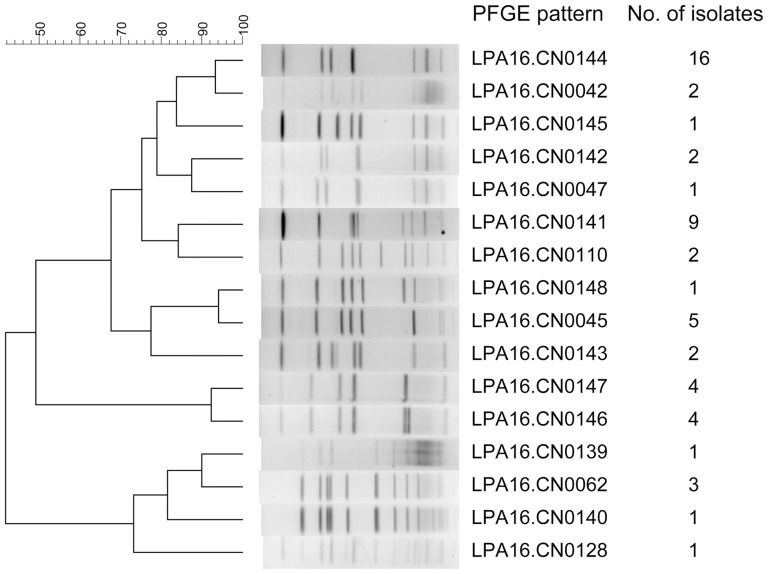
Clustering results of patterns obtained by PFGE analysis of 55 *Legionella* strains. These strains were isolated from one sample of hot spring recreational area A, which was collected in April.

To further show the high genetic polymorphism of strains that were isolated from the spa water samples, we selected another 55 isolates from one sample from area A that was collected in April. These 55 isolates were divided into 16 different patterns with one to sixteen in each pattern (Figure 4). The pattern LPA16.CN0144 was dominant and contained sixteen (29.1%) isolates. Among these sixteen patterns, nine occurred during other months in area A, and five occurred in other areas. However, the second-most dominant pattern LPA16.CN0141 only occurred in area A in April.

For SBT, 57 *L. pneumophila* isolates were selected based on their PFGE patterns and serogroups and isolated, and these isolates were differentiated by SBT into 35 different sequence types (STs). Ten profiles (ST59, ST81, ST87, ST246, ST354, ST377, ST595, ST961, ST1101 and ST1279) could be found in the EWGLI SBT database, but the profile of ST-CN1 to ST–CN25 could not be found in the database.

**Figure 5:**
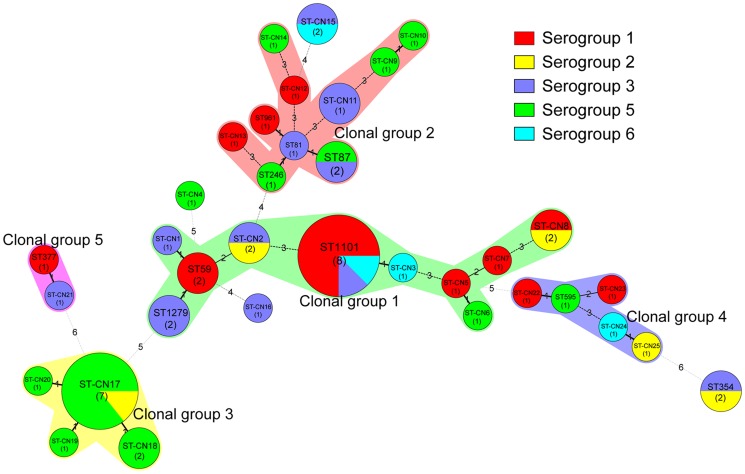
Minimum spanning tree analysis of 57 *Legionella* isolates from hot springs in Beijing, China. In the minimum spanning tree, the STs are displayed as circles. The size of each circle indicates the number of isolates within this particular type, and the STs and the number of isolates in each ST are shown in the circles. The colors of the halo surrounding the STs denote types that belong to the same clonal group.

According to the results of our eBURST analysis, 35 STs belonged to 5 CGs and 4 singleton STs (Table 3, Figure 5). Of the 5 CGs, CG1, which was the prevalent clonal group, included 21 isolates that belonged to ST59, ST1101, ST1279, ST-CN1, ST-CN2, ST-CN3, ST-CN5, ST-CN6, ST-CN7 and ST-CN8; and the putative ancestor of CG1 was predicted to be ST59. The reminders were CG2 (ST81, ST87, ST-246, ST-961, ST-CN9, ST-CN10, ST-CN11, ST-CN12, ST-CN13 and ST-CN14), which contained 12 isolates, CG3 (ST-CN17, ST-CN18, ST-CN19 and ST-CN20), which contained 11 isolates, CG4 (ST595, ST-CN22, ST-CN23, ST-CN24 and ST-CN25), which contained 5 isolates and CG5 (ST377 and ST-CN21), which contained 2 isolates. Of the 4 singleton STs, ST354 and ST-CN15 were detected in 2 isolates.

**Table 3:** Distribution of clonal groups from 35 SBT profiles for *L. pneumophila* isolates (n = 57) in hot springs in Beijing, China.

Clonal groups (CGs)	STs	*flaA*	*pilE*	*asd*	*mip*	*momps*	*proA*	*neuA*	No. of isolates
CG1 (n = 21)	ST-59	7	6	17	3	13	11	11	2
	ST-1101	6	6	15	3	9	14	11	8
	ST-1279	7	6	17	3	2	11	11	2
	ST-CN1	7	6	17	3	13	103	11	1
	ST-CN2	2	6	17	3	9	11	11	2
	ST-CN3	6	6	15	3	9	14	2	1
	ST-CN5	6	10	17	3	2	14	2	1
	ST-CN6	17	10	17	3	2	14	2	1
	ST-CN7	2	10	17	101	2	14	2	1
	ST-CN8	2	10	17	14	21	14	0	2
CG2 (n = 12)	ST-81	2	10	3	28	9	4	9	1
	ST-87	2	10	3	28	9	4	13	2
	ST-246	2	10	3	28	9	4	11	1
	ST-961	2	10	3	28	9	14	9	1
	ST-CN9	17	10	15	28	21	4	207	1
	ST-CN10	17	10	15	28	21	4	2	1
	ST-CN11	6	10	15	28	4	4	9	2
	ST-CN12	2	10	15	28	19	4	3	1
	ST-CN13	2	0	15	28	21	4	11	1
	ST-CN14	2	43	15	18	19	4	11	1
CG3 (n = 11)	ST-CN17	8	6	34	9	2	8	209	7
	ST-CN18	8	6	34	9	2	8	9	2
	ST-CN19	8	6	7	9	2	8	209	1
	ST-CN20	8	6	34	9	2	102	209	1
CG4 (n = 5)	ST-595	2	14	16	16	15	13	2	1
	ST-CN22	6	14	16	16	15	13	2	1
	ST-CN23	2	14	16	31	15	13	210	1
	ST-CN24	11	14	16	28	15	13	3	1
	ST-CN25	11	14	16	7	15	13	3	1
CG5 (n = 2)	ST-377	10	22	7	28	16	18	6	1
	ST-CN21	10	22	34	28	16	18	6	1
Singletons (n = 6)	ST-354	3	5	1	7	14	32	8	2
	ST-CN4	32	6	48	6	48	101	11	1
	ST-CN15	2	6	14	28	21	8	3	2
	ST-CN16	7	6	17	28	101	14	3	1

### Intracellular growth ability

**Figure 6:**
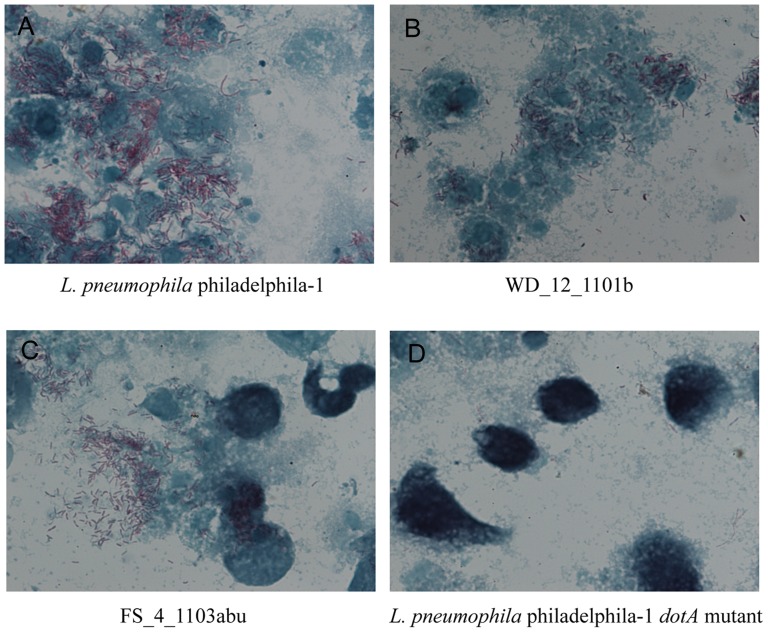
Gimenez staining and light microscope observation of J774 cells after infection by *Legionella*. The photos were captured on day 2 of infection. The *L. pneumophila* philadelphila-1 strain (A) and its *dotA* mutant (D) were used as positive and negative controls, respectively. We selected isolates WD_12_1101b (B) and FS_4_1103abu (C) as representative strains of the 57 tested strains. J774 cells were stained with malachite green (green), and the bacteria were stained with fuchsin (dark red).

**Figure 7:**
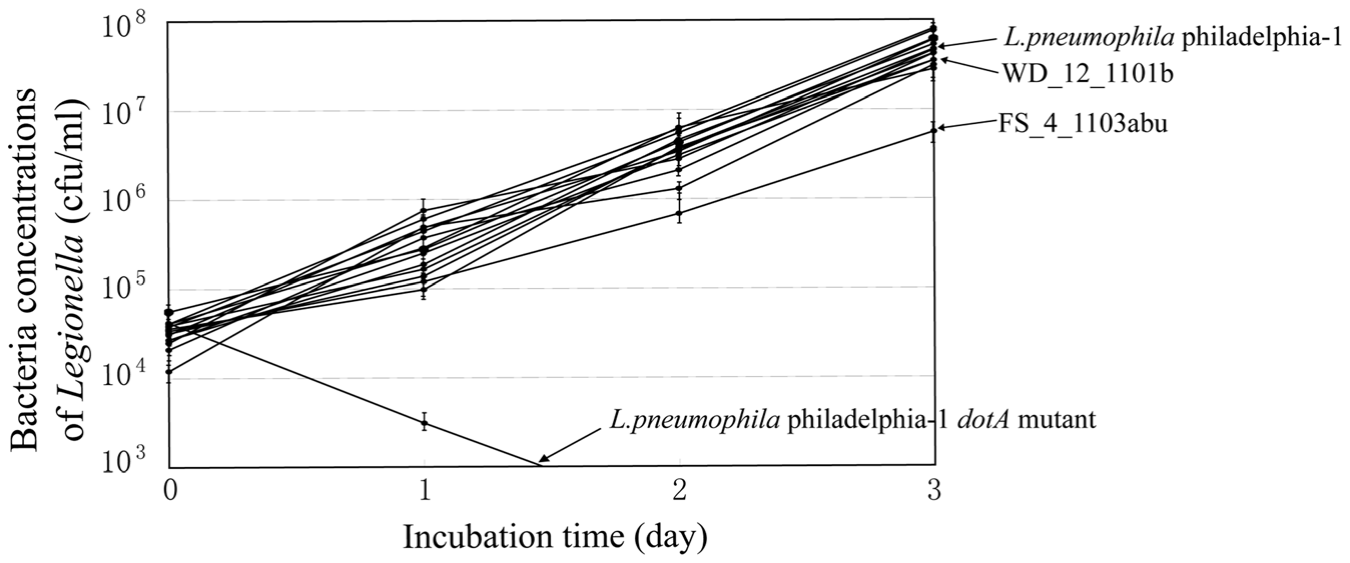
Intracellular growth of *Legionella* isolates within J774 cells. The isolated *Legionella* strains were grown in BYE broth until they reached early stationary phase. Approximately 2×10^9^ bacteria diluted (1∶1,000) in RPMI 1640 tissue culture medium were added to J774 cells (2×10^5^ per well) with a multiplicity of infection (MOI) of approximately 10. The infected cells were incubated at 37 °C under 5% CO_2_-air for 1.5 h and washed three times with PBS to remove extracellular bacteria. The CFUs were determined by plating dilutions on BCYE agar plates. The *L. pneumophila* philadelphila-1 strain and its *dotA* mutant were used as positive and negative controls, respectively.

The 57 isolates that were analyzed by SBT were also tested for their intracellular growth ability. For all of the isolates, intracellular bacteria could be observed under a light microscope by Gimenez staining on day 2 of J774 cell infection, which suggested that all isolates have intracellular growth ability in J774 cells (Figure 6). To further evaluate the intracellular growth ability of the isolates, plate counting was performed. All isolates, except isolate FS_4_1103abu, had bacterial concentrations of 10^4^–10^5^ CFU/ml, 10^5^–10^6^ CFU/ml, 10^6^–10^7^ CFU/ml and 10^7^–10^8^ CFU/ml on day 0, 1, 2 and 3 of infection, respectively, and showed no difference in bacterial concentrations of *L. pneumophila* philadelphia-1 by the t test (p>0.05). FS_4_1103abu exhibited bacterial concentrations of 6.8×10^5^ CFU/ml and 5.6×10^6^ CFU/ml on day 2 and 3 of infection, respectively, and was significantly different from that of *L. pneumophila* philadelphia-1 ((p<0.05). However, FS_4_1103abu still showed high proliferation ability in J774 macrophage cells (Figure 7).

## Discussion

This study illustrates a high degree of pollution of *Legionella* in hot spring water samples in Beijing, China. *Legionella* were detected in 51.9% of collected hot spring water samples from three hot spring recreation areas during the one-year study period. The isolation rate in this study was higher than what was reported by previous studies in Japan (28.7%) [32] and Taiwan (27.8%) [33]. The positive rate showed the severity of contamination of hot spring water in Beijing. If culture methods were utilized that might underestimate the presence of *Legionella* species, the actual level of contamination could be greater. The concentration of *Legionella* was low in this study, with only 7.5% of the samples giving a *Legionella* concentration that exceded 1000 CFU/liter, which was lower than what was found in Japan [32]. In the study carried out in Japan, nearly half of the samples exhibited *Legionella* concentrations that exceeded 1000 CFU/liter. The above findings clarified that although the number of the bacteria was low, *Legionella* widely and long-standingly inhabits hot spring water in Beijing.

In this study, the *Legionella* pollution and concentrations at three different hot spring recreational areas differed. These results were consistent with those obtained in other studies [32], [33]. The *Legionella* pollution and concentration were also different between seasons in this study: the levels were higher in spring and winter but lower in summer and autumn. In another study that looked at cooling towers in China, the highest isolation rate and concentration was observed in autumn and showed a positive association between *Legionella* presence in cooling towers and increasing air temperatures [15]. However, in this study, the association between the presence of *Legionella* in spring water samples and increasing air temperatures was negative. Because the air temperature could significantly impact the water temperature of cooling towers but not that of spring water, we speculate that no association between the presence of *Legionella* in spring water samples and air temperatures will occur. Another factor, passenger flow, may explain the differences in the presence of *Legionella* between seasons. Spring and winter were the peak periods for Chinese to visit hot spring recreational areas, and a positive association between the presence of *Legionella* and the increasing passenger flow was shown in this study. Based on our results, we suggest that more disinfection measures are needed in spring and winter than in summer and autumn.

The growth and survival of all organisms is constrained by specific ranges of physicochemical parameters. The temperature and pH of water samples have been considered major impact factors of contamination by *Legionella* [34]. However, in this study, no significant association was found between the *Legionella* positive rate and sample temperature ≥40 °C or pH≥7.9. We believe that this is due to the small interval of temperature (31 °C to 49 °C) and pH (7.80 to 8.04) of the spring water samples that were collected in this study. *Legionella* are able to reproduce at temperatures between 25 and 50 °C and survive in temperatures of up to 55–60 °C; therefore, they thrive easily in naturally and artificially hot water systems [35]. It is known that *Legionella* easily reproduce in high temperature water systems, and an inverse association between water temperature and the concentration of *Legionella* was found [36], [37], [38]. In a previous study, *Legionella* species were found in water with temperatures ranging from 23.7 °C to 60.3 °C, and a significant association between the *Legionella* positive rate and sample temperature of≥55 °C was found [34]. However, the highest sample temperature (49 °C) in this study was lower than 55 °C, and no significant difference between the *Legionella* positive rate that was found between 31–40 °C and 41–49 °C were found in the present study. In Furuhata's study, the isolation rates were significantly different when the pH was lower or higher than 3. The pH of all spring water samples in this study was near 8.0, which was an optimal pH for *Legionella* in an aquatic environment [20], [33], [37]. The free chlorine concentration is another important impact factor for contamination of spring water by *Legionella*. Mouchtouri et al. found that *Legionella* colonization was positively associated with a free chlorine concentration of ≥0.2 mg/L, which is in agreement with the results of the present study [34]. The microbiological parameters of spring water that were examined here included the total microbial counts and total coliform, and contamination by *Legionella* was associated with total microbial counts and total coliform levels in samples that were collected from the same water distribution system. This association may be explained by the ecological water environment being suitable for colonization and reproduction of *Legionella* and it being beneficial for other microorganisms. Therefore, the total microbial counts and total coliform could be used as indicators of the presence of *Legionella*.

*L. pneumophila* was the major species that was isolated in this study, and this finding was in agreement with studies of spring water in other countries and other types of water samples in China. *L. pneumophila* accounted for 85.7% and 78.6% of the strains that were isolated from spring waters in Japan [39] and South Korea [14], and this organism was predominantly found in other types of water samples, such as water from buildings, public baths, hospitals, factories and hotels. In the South Korean study [14], *L. pneumophila* serogroup 6 was the most frequently isolated serogroup, but it was unreasonable to conclude that serogroup 6 predominated in springs because only 14 isolates were collected from springs in that study. In the Japan study [39], serogroups 1, 5 and 6 were the predominate serogroups accounting for 22%, 21% and 22% of positive samples respectively. In this study, serogroups 3, 6, 5 and 1 were determined to be the predominate serogroups in springs and accounted for 25.3%, 23.4%, 19.2% and 18.5%, respectively. *L. pneumophila* serogroup 1 was the predominate serogroup in cooling tower water, as another study reported [15]. However, we found that *L. pneumophila* serogroup 1 was not predominant in springs in this study. Non-pneumophila infections, *L. micdadei*, *L. bozemanii*, *Legionella dumoffii*, *Legionella longbeachae* and other species are the cause of most of the reported infections [40], [41], [42]. In this study, we isolated one *L. bozemanii* and two *L. micdadei* serotypes from spring water. *L. micdadei* was the second-most dominant strain in cooling tower water from Shanghai, China [15]. Therefore, non-*L. pneumophila* species, especially *L. micdadei*, should also be taken into account when a Legionnaires' disease is being suspected in a clinical setting.

We were surprised at the variety of species and *L. pneumophila* serogroups and of the many different PFGE patterns of strains that were isolated from the same water samples. We selected 1–10 strains from one spring recreation area within one month to be analyzed by PFGE to describe the population structure of *Legionella* in springs. The results showed high genetic polymorphism of the tested strains. To describe the complex population structure of *Legionella* in springs, 55 isolates from one sample were analyzed by PFGE. This analysis showed that although high genetic polymorphism was displayed, a dominant pattern also existed in springs. The same patterns persisted in the investigated spring recreation area throughout the one-year study period.

SBT results showed that the population of STs was highly diverse. Fifty-seven isolates were divided into 35 STs, of which 25 STs (ST-CN1 to -CN25) had new allelic profiles. After querying the SBT database, we found six STs (ST59, ST81, ST87, ST354, ST595 and ST961) that had previously caused Legionnaires' diseases worldwide. In our previous study, we reported four Legionnaires' diseases that were caused by strains of ST36 and ST346 [43]. However, no isolates belonging to these two STs were detected in this study. In 2011, we detected a case of Legionnaires' diseases caused by a *L. pneumophila* strain of ST59 (data not published) in Beijing; however, no direct epidemiological relationship was found between this case and that of the hot springs.

Invasion and intracellular replication of *Legionella* within protozoa in the environment play major roles in the transmission of Legionnaires' disease, and important relationships between the intracellular growth ability of *Legionella* within protozoa and macrophages and human legionellosis have been seen [44], [45]. Theoretically, *Legionella* isolates with invasive and intracellular growth ability can infect the human body. In this study, all isolates that were studied using the intracellular growth assay could enter a macrophage and had high intracellular growth ability, which suggests that these hot spring isolates have the potential to cause human legionellosis.

In conclusion, our results demonstrated high prevalence and genetic polymorphism of *Legionella* in springs in Beijing, China. Furthermore, the SBT and intracellular growth assay results suggested that the *Legionella* isolates of hot spring environments were pathogenic. Because of the potential for spring water systems to harbor, amplify and disseminate *Legionella*, improved control and prevention strategies are urgently needed.
